# Acute promyelocytic leukemia (APL): a review of the literature

**DOI:** 10.18632/oncotarget.27513

**Published:** 2020-03-17

**Authors:** Joaquin J. Jimenez, Ravinder S. Chale, Andrea C. Abad, Andrew V. Schally

**Affiliations:** ^1^Dr. Phillip Frost Department of Dermatology, Miller School of Medicine, University of Miami, Miami, FL, USA; ^2^Department of Biochemistry and Molecular Biology, Miller School of Medicine, University of Miami, Miami, FL, USA; ^3^Division of Endocrinology, Department of Medicine, Miller School of Medicine, University of Miami, Miami, FL, USA; ^4^Endocrine, Polypeptide and Cancer Institute, Veterans Affairs Medical Center, Miami, FL, USA; ^5^Department of Medicine, Sylvester Comprehensive Cancer Center, Miller School of Medicine, University of Miami, Miami, FL, USA; ^6^Division of Hematology Oncology, Department of Medicine, Miller School of Medicine, University of Miami, Miami, FL, USA; ^7^Department of Pathology, Miller School of Medicine, University of Miami, Miami, FL, USA

**Keywords:** promyelocytic leukemia, arsenic trioxide, retinoic acid, resistance

## Abstract

Acute Promyelocytic Leukemia (APL) is characterized by a block in differentiation where leukemic cells are halted at the promyelocyte stage. A characteristic balanced chromosomal translocation between chromosomes 15 and 17 t (15;17) (q24; q21) is seen in 95% of cases — the translocation results in the formation of the PML-RARA fusion protein. The introduction of retinoic acid (RA) and arsenic trioxide (ATO) has been responsible for initially remarkable cure rates. However, relapsed APL, particularly in the high-risk subset of patients, remains an important clinical problem. In addition, despite the success of ATRA & ATO, many clinicians still elect to use cytotoxic chemotherapy in the treatment of APL. Patients who become resistant to ATO have an increased risk of mortality. The probability of relapse is significantly higher in the high-risk subset of patients undergoing treatment for APL; overall approximately 10-20% of APL patients relapse regardless of their risk stratification. Furthermore, 20-25% of patients undergoing treatment will develop differentiation syndrome, a common side effect of differentiation agents. Recent evidence using *in vitro* models has shown that mutations in the B2 domain of the PML protein, mediate arsenic resistance. Alternative agents and approaches considering these clinical outcomes are needed to address ATO resistance as well as the relapse rate in high risk APL.

## INTRODUCTION

In recent decades, treatment of Acute Promyelocytic Leukemia (APL) has served as a representation of targeted therapy and has reflected the power of translational research. APL was first reported and described by Norwegian hematologist, LK Hillestad in 1957. In 1959, J. Bernard identified 20 patients with APL, providing an even more extensive description, including its connection to the proliferation of promyelocytes, catastrophic hemorrhagic incidences, and hyper-acute onset [1]. APL accounts for 10%–15% of all acute myeloid leukemia’s [2]. The disease is classified according to the French-American-British (FAB) classification system as AML-M3. APL is characterized by a block in differentiation where leukemic cells are halted at a distinct stage in cellular maturation, specifically the promyelocyte stage. A characteristic balanced chromosomal translocation between chromosomes 15 and 17 t (15;17) (q24; q21) is seen in 95% of cases, which results in the expression of the Promyelocytic leukemia (PML)–retinoic acid receptor-alpha (RARA) fusion protein, collectively known as PML-RARA [3]. The PML-RARA fusion protein yields a dominant negative mutation, blocking differentiation while simultaneously preventing apoptosis and enabling the proliferation of leukemic progenitors [4]. The fusion protein has been at the center of many *in vitro* studies over the past few decades enabling a better understanding of the molecular biology behind APL as well as its unique response to retinoic acid.

The introduction of all-trans retinoic acid (ATRA), as well as of arsenic trioxide (ATO) in the treatment of APL, was crucial to achieving the current remarkable cure rates. As opposed to the traditional cytotoxic chemotherapeutic agents conventionally used in the treatment of various cancers, ATRA, as well as ATO at low doses, are differentiating agents. The initial evidence of the differentiating properties of retinoic acid and its potential to be used therapeutically came in 1980, first using the HL-60 cell line as a model for APL [5]. At the time HL-60 was characterized as AML-M3 since it expressed a promyelocytic phenotype. This classification was later revised and HL-60 is now characterized as AML-M2 in the updated classifications. Nonetheless, Breitman et al. provided the first evidence that ATRA could cause promyelocytes to differentiate into fully mature granulocytes [5]. Shortly after the introduction of retinoic acid into the therapy regimen of APL, the need arose for addressing retinoic acid resistance. Resistance to ATRA was partially alleviated by the advent of arsenic trioxide; however, treatment resistance still remains an issue to this day. APL has been plagued by an abnormally high early death rate as well as bleeding complications [6, 7]. Furthermore, up to 50% of patients undergoing treatment will develop differentiation syndrome; a common side effect of differentiating agents [8].

Typically, APL patients can be risk-stratified into three groups- low, intermediate, and high according to WBC counts, [9]. The low and intermediate subset of patients may be grouped together and are defined by a WBC of less than 10,000/µL [9]. High-risk patients are defined as having a WBC above 10,000/µL [9]. Although intermediate as well as low-risk patients may be treated without the use of cytotoxic chemotherapy, the combination of ATRA and ATO alone is not sufficient to treat high-risk patients [10]. The treatment of high-risk patients, (defined as having a WBC count greater than 10,000/µL)- involves administration of cytotoxic chemotherapy [10]. An evaluation of four clinical trials involving low risk APL patients (WBC count ≤ 10 × 10^9^/L) from 2010–2014 showed overall survival rates (%) ranging from a low of 86% after three years to a high of 99% after 4 years [11–14]. In contrast, evaluation of three clinical trials from 2015–2017 involving high risk APL patients (WBC count > 10 × 10^9^/L) showed overall survival rates ranging from a high of 88% after 3 years to a low of 86% after 5 years [15–17]. The probability of relapse is significantly higher in the high-risk subset of patients undergoing treatment for APL; however, approximately 10–20% of APL patients relapse regardless of their risk stratification [18].

## MOLECULAR BASIS AND TREATMENT OF APL

**Figure 1:**
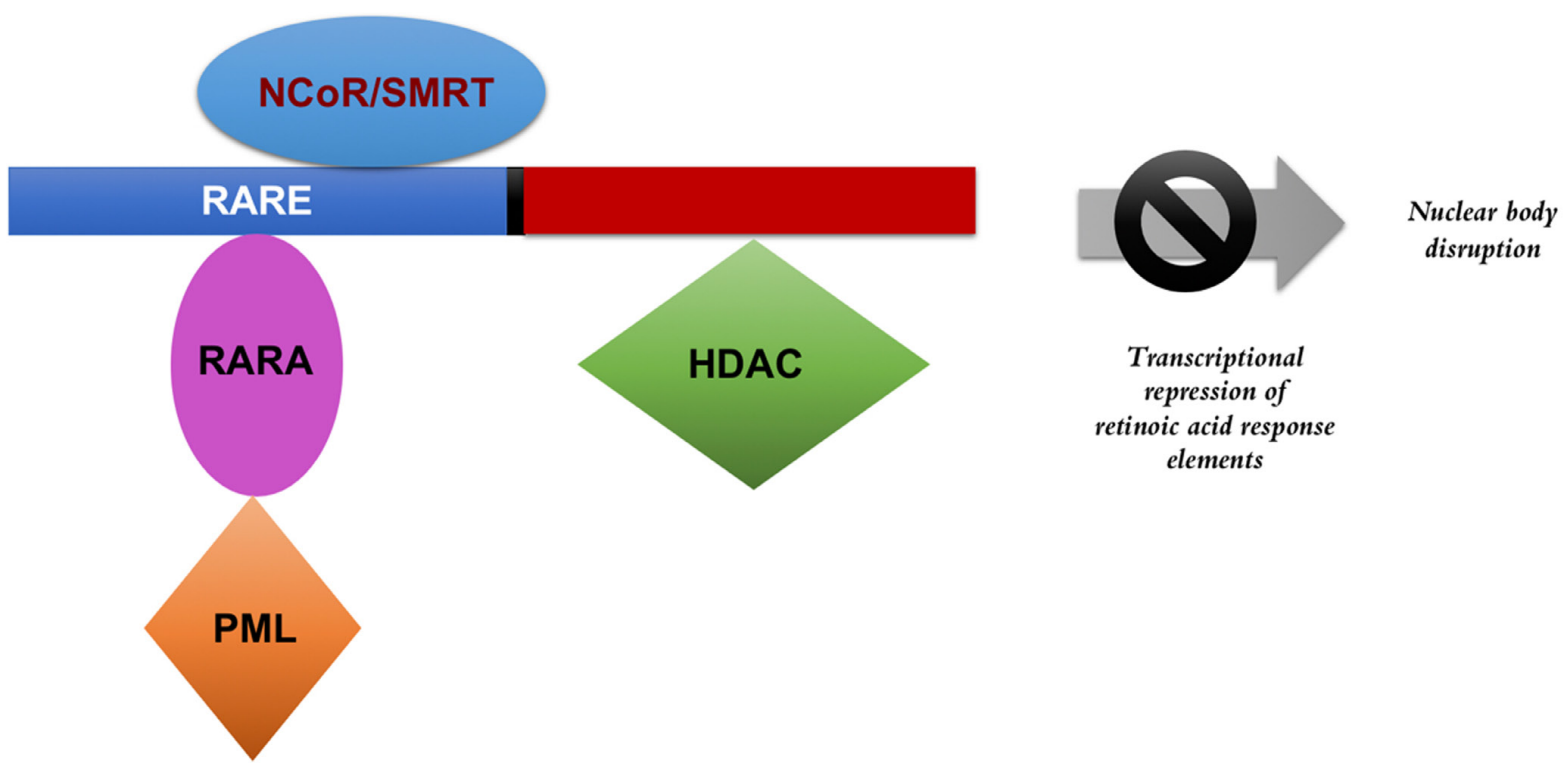
PML-RARA transcriptional repression. The presence of the fusion protein interferes with the transcription of retinoic acid response elements and disrupts the formation of nuclear bodies. The fusion protein, in the absence of pharmacological doses of retinoic acid, recruits co-repressors to silence gene transcription related to differentiation and prevents apoptosis. NCOR: nuclear receptor corepressor, SMRT: silencing mediator for retinoid and thyroid hormone receptor, RARE: retinoic acid response elements, RARA: retinoic acid receptor alpha, PML: promyelocytic leukemia protein, HDAC: histone deacetylase.

The molecular basis behind APL has been largely focused on the role of the PML-RARA fusion protein. PML-RARA interferes with gene expression of hematopoietic progenitor self-renewal as well as with myeloid differentiation [19]. In normal cells, the retinoic acid receptor alpha (RARα) forms a heterodimer with another form of a nuclear hormone receptor protein called retinoid X receptors (RXR) [20]. Together, the RARα-RXR heterodimer binds to regions of DNA referred to as retinoic acid response elements (RAREs) to mediate the transcription of hundreds of genes. Many of the RAREs are involved in self-renewal and differentiation. In the absence of ligand (retinoic acid) binding, the heterodimeric protein complex recruits corepressors such as the nuclear receptor corepressor (NCoR) and the silencing mediator for retinoid and thyroid hormone receptor (SMRT) [21] (Figure 1). Additional proteins and enzymes involved in transcriptional repression, particularly histone deacetylases (HDAC), are also recruited. The binding of retinoic acid to the ligand-binding domain of RARα triggers a conformational change, enabling the protein complex to release corepressors and begin to recruit coactivators. Subsequently, chromatin remodeling occurs, along with the recruitment of transcriptional machinery leading to gene expression [21]. According to the classical model of APL pathogenesis, fusion of the PML and RARA proteins disrupts this coactivator recruitment, preventing transcription of retinoic acid response elements.

However, recent evidence has emerged painting a much more complex picture of APL pathogenesis. For example, PML-RARA tetramers have the ability to bind to a large number of target DNA sites not recognized by the normal RARα-RXR receptors [22]. The ability of the fusion protein to recognize unique non-canonical DNA sites may contribute to widespread transcriptional deregulation [22].

Furthermore, the PML-RARA fusion protein retains crucial functional domains that contribute to leukemogenesis as well as sensitivity to retinoic acid and arsenic trioxide [23, 24]. For RARα, these domains include the DNA binding domain, hormone binding domain, and the RXR-binding domain [23, 24]. PML retains the coiled-coil and the RING finger domains [23, 24].

The PML-RARA fusion protein also disrupts the formation of PML nuclear bodies, a process that has been implicated in the transformation of APL cells [19]. PML nuclear bodies are sphere-shaped domains that localize to the nuclear matrix and are known to be involved in many functions associated with the nucleus such as epigenetic silencing, transcription, and replication [25]. PML nuclear domains also modulate p53 signaling, as well as senescence, possibly through their ability to control sumoylation and proteolysis [26]. The PML protein is the key organizer of these domains and is important in the formation of nuclear bodies [25]. PML has been shown to recruit a number of partner proteins, one of the most important being DAXX, a repressor of transcription and modulator of apoptosis [25]. The PML protein itself undergoes several posttranslational modifications, such as phosphorylation and sumoylation [25]. The protein’s ability to be sumoylated plays a critical role in the recruitment of partner proteins [25].

Chromatin immunoprecipitation (ChIP)-sequencing studies have shown that the RXRA usually co-localizes at PML-RARA bound DNA promoter regions [22, 27]. Presence of RXRA in the vicinity of PML-RARA has shown to enhance the latter’s DNA binding ability [28]. The presence of a PML-RARA-RXR complex has been associated with APL pathogenesis [29]. The sumoylation of RXRA plays a significant role in the transformation of APL cells [19].

Treatment of APL cells with pharmacological doses of retinoic acid induces blast differentiation as well as the degradation of the fusion protein [30, 31]. Inhibition of RARα may involve a normal negative feedback loop of RA on its own receptors [32, 33]. In addition, treatment of APL cell lines with retinoic acid is believed to alleviate a repressive chromatin environment, allowing transcriptional activators to bind to retinoic acid response elements [30, 34].

**Figure 2:**
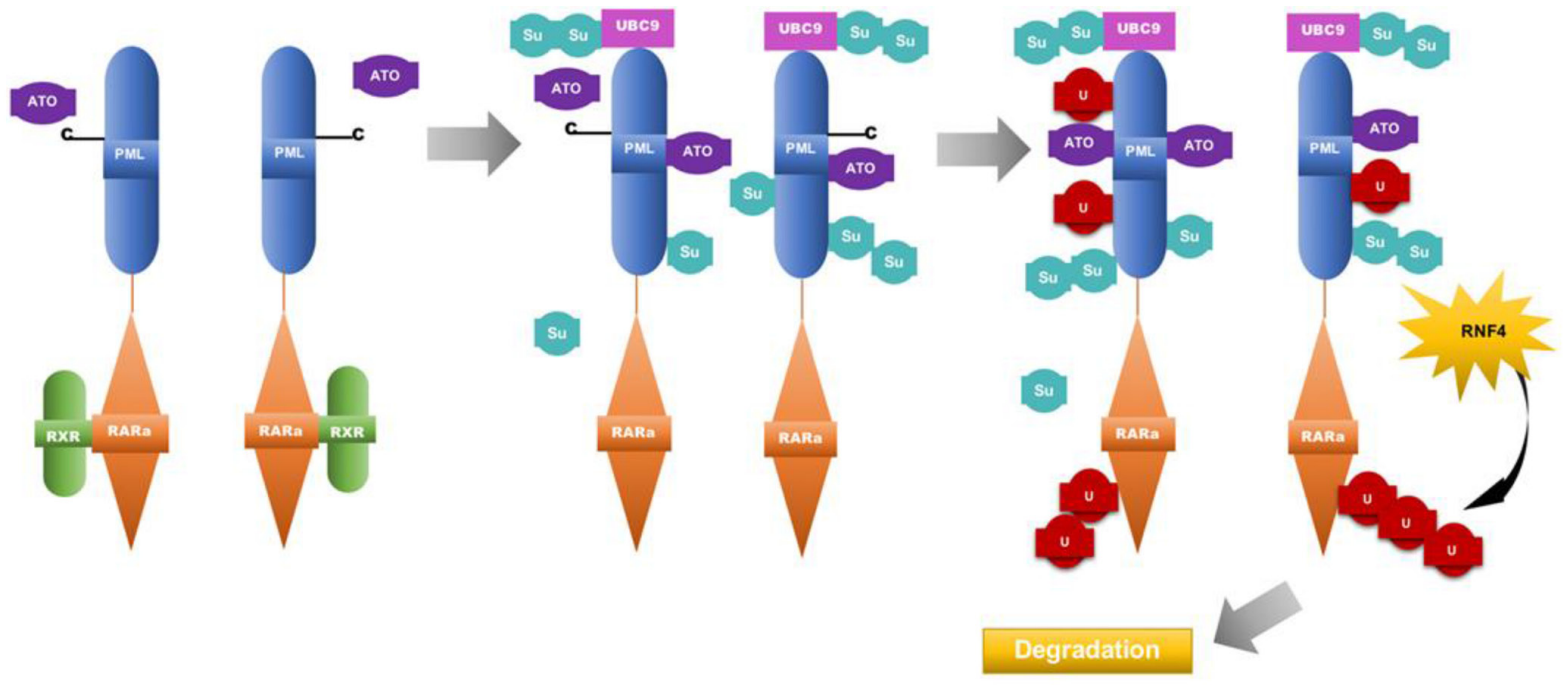
Model for the mechanism of Arsenic trioxide in APL therapy. Arsenic trioxide binds to cysteine residues on the PML moiety of PML-RARA, triggering the binding of ubiquitin-conjugating enzyme 9 (UBC9) to the PML RING finger domain. UBC9 recruitment then allows the PML-RARA moiety to undergo sumoylation [19]. The attachment of these ubiquitin-like proteins recruits ring finger protein 4(RNF4) onto PML nuclear bodies [25, 35, 36]. RNF4 is a SUMO-dependent ubiquitin ligase that polyubiquitylates PML, targeting it towards the proteasome for degradation. ATO: arsenic trioxide, RXR: retinoic X receptor, U: ubiquitin molecules, Su: SUMO groups, RNF4: ring finger protein 4.

Arsenic trioxide treatment of APL cells can induce degradation of both the PML-RARA fusion protein and the normal PML protein [19]. ATO has been shown to bind to cysteine residues on the PML moiety of the fusion protein while targeting it towards nuclear bodies [19]. The binding of ATO triggers or enhances the binding of ubiquitin-conjugating enzyme 9 (UBC9) to the PML RING finger domain [19]. UBC9 recruitment then allows the PML-RARA moiety to undergo sumoylation [19]. Subsequently, the attachment of these ubiquitin-like proteins recruits ring finger protein 4 (RNF4) onto PML nuclear bodies [19, 35, 36]. RNF4 is a SUMO-dependent ubiquitin ligase that polyubiquitylates PML, targeting it towards the proteasome for degradation [19, 35, 36] (Figure 2). Thus, the primary mechanism behind arsenic trioxide-induced remission involves the targeting of the PML-RARA or normal PML towards nuclear bodies. Arsenic trioxide also enhances the reformation of nuclear bodies. Both retinoic acid and arsenic trioxide, agents that operate by different mechanisms, are capable of inducing PML-RARA degradation; which contributes significantly to disease remission.

Interestingly, the presence of the PML-RARA fusion protein is a requisite that confers sensitivity to RA; however, increased degradation through proteasomal pathways of PML-RARA may be contributing to retinoic acid resistance [37]. Retinoic acid resistant cell lines such as NB4.007/6 show little to no detectable levels of PML-RARA transcripts, and the loss of PML-RARA expression is due to increased degradation through the proteasome pathway in this particular cell line [37]. Thus, the presence of the PML-RARa protein serves a dual role: Its expression leads to the initiation of APL while its presence is necessary to confer sensitivity to pharmacological doses of retinoic acid. Given that the HL-60 cell line does not possess the characteristic translocation t (15;17), it provides an important model for studying resistance to therapy. Several ATRA-resistant HL-60 cell lines have been isolated [38, 39]. *In vitro* studies on HL-60 show that aberrant signaling mechanisms involving C-Raf, Vav-1 and Fgr during early and late differentiation may be responsible for resistance [38]. Exposure to compounds such as vitamin D3 partially restores responsiveness to ATRA [38]. These studies suggest that exploring the use of other nontoxic differentiating agents, in combination with RA may provide a route to overcoming resistance.

## MUTATIONAL LANDSCAPE OF APL

Aside from the expression of the characteristic PML-RARA fusion protein, APL has a unique mutational landscape in regards to other somatic co-mutations. Recurrent mutations have also been noted in FLT3, WT1, NRAS, KRAS, ARID and PML genes [40]. The Fms like tyrosine kinase 3 (FMLT3) gene has been shown to be the most commonly mutated in primary APL [40]. Non-silent mutations may provide a cooperative effect with the PML-RARA fusion in disease progression. The genetic alterations that occur in APL differ at diagnosis and at relapse. Mutations in PML and RARA proteins are common in relapse APL and are rarely seen at initial diagnosis [40]. An increased in the frequency of mutations was also noted in ARID1B and RUNX1 genes in relapse [40]. Additionally, other commonly known AML mutations such as NPM1 and DNMT3A are not seen in APL [40].

### Pin 1 and its role in APL

Proline-directed phosphorylation (pSer/Thr-Pro) has been shown to play a role in many cellular signaling pathways, some of which are involved in oncogenesis [41]. Pin-1 is a Peptidyl-prolyl cis/trans isomerase (PPIase) that recognizes and isomerizes the specific phosphorylated Ser/Thr-Pro (pSer/Thr-Pro) chain of amino acids in various proteins [42, 43]. Isomerization usually leads to conformational changes in a large subset of proteins that can act as molecular switches, enabling control of many cellular signaling pathways virtually by a single phosphorylation event [41–43]. The amino acid proline can adopt cis/trans conformations; most of these isomerizations are controlled by prolyl isomerases (PPIase) including Pin-1 [41]. Pin-1 has often shown to be overexpressed in many human cancers, due to its ability to simultaneously activate oncogenes and inhibit tumor supressors [41–43]. Wei et al., have demonstrated that Pin-1 is a direct target of ATRA [41]. Binding of ATRA to Pin-1 ultimately leads to its destruction [41]. ATRA possess a carboxyl group which can form salt bridges with critical basic residues in the Pin-1 substrate binding site [41]. The carboxyl moiety in ATRA essentially mimics the pSer/Thr-Pro targets that Pin-1 recognizes [41]. In addition, the aromatic groups of ATRA bind to the active site of Pin-1, rendering it ineffective [41].

Interestingly, many studies have shown the ability of ATRA to induce degradation of PML-RARA and inhibit self-renewal, while being decoupled from its ability to activate the retinoic acid receptor-α. This suggests that activation of the nuclear hormone receptor may not be necessary for APL remission [44, 45]. *In vitro* studies involving the APL cell line NB4 have shown that inactivating retinoic acid receptors does not prevent ATRA from inducing Pin-1 degradation, nor does this affect its ability to inhibit NB4 cell growth [41]. Thus, Pin-1 may be an attractive target for future studies, involving cases that have become resistant to the standard ATRA-ATO treatment combination.

## THE ROLE OF AUTOPHAGY IN APL

Several recent studies have suggested a role of autophagy in APL pathogenesis. Autophagy refers to a highly conserved degradative process by which intracellular compartments and materials are recycled or removed [46, 47]. During the process, membrane bound vesicles (autophagosomes) containing intracellular materials fuse with the lysosome for degradation [46, 47]. The process is largely regulated by a set of autophagy-related genes (ATG’s) [46]. Several signaling pathways have been shown to control the expression of ATG’s including the mechanistic target of rapamycin kinase (MTOR) and AMP-activated protein kinase (AMPK) pathways [48, 49]. Decreased expression of ATG’s has been noted in primary AML blasts [50]. ATRA-induced differentiation has been shown to increase the expression of ATG’s as well as restore the process of autophagy in NB4 and Hl-60 cell lines [50, 51]. The p62 or sequestosome 1 (p62/SQSTM1) adapter protein plays a crucial role in directing ubiquitinated proteins towards autophagosomal vesicles [52]. Introduction of ATRA has shown to increase mRNA levels of p62/SQSTM1 in a Nf-kB dependent manner during differentiation [52]. Upregulation of autophagy may confer a survival advantage that allows mature granulocytes to complete differentiation while limiting non-essential aggregate proteins [52].

Along with other major genetic alterations (PML-RARA), the disruption of autophagy in APL may halt differentiation and contribute to the proliferation of promyelocytes [52–54]. The upregulation of autophagy and its associated genes may prove to be useful in therapy. Treatment of APL cells with arsenic trioxide has been shown to upregulate ATG’s via the mTOR pathway, suggesting that autophagy plays a role in the degradation of the PML-RARA oncoprotein [55]. Therapy induced differentiation was also correlated with increased autophagy in APL cells [55].

Beclin-1 has long been known to play a central role in autophagy. Beclin-1 forms part of the PI3K complex that is involved in the initiation and maturation of autophagosomes [56, 57]. Both ATRA and ATO therapy has shown to increase autophagy via the upregulation of Beclin-1 [56, 57].

### Treatment complications of APL

The differentiation syndrome is the most common and potentially life-threatening complication associated with the treatment of APL [8]. The differentiation syndrome (also known as retinoic acid syndrome) commonly occurs within one to two weeks after initiation of ATRA and/or ATO [8, 58]. Clinical manifestations associated with this side effect include dyspnea, pulmonary inðltrates, unexplained fever, pleuro-pericardial effusion, hypotension, acute renal failure, and peripheral edema [8, 58, 59]. Close to 50% of patients undergoing treatment with ATRA will develop differentiation syndrome [8, 10, 58]. Patients who experience ATRA syndrome have a significantly lower event-free survival (EFS) and overall survival (OS) compared to patients who do not develop this complication of treatment [8, 9]. The exact molecular mechanisms of how differentiation syndrome develops as a result of treatment are not fully understood. However, exposing APL blasts to pharmacological doses of ATRA *in vitro* triggers biological responses reminiscent of differentiation syndrome [8]. *In vitro* studies suggest that the development of differentiation syndrome may be due to changes in the cytokine secretion as well as certain adhesive qualities of APL cells during ATRA-induced differentiation [60]. ATRA may up-regulate the expression of certain adhesion molecules on the surface of APL cells. In particular, the expression of high-affinity β2 integrins such as leukocyte function-associated antigen-1 (LFA-1) or the type I transmembrane glycoprotein intercellular adhesion molecule (ICAM-2) is increased *in vitro* when NB4 promyelocytes are exposed to ATRA [60]. Expression of these adhesion molecules leads to the aggregation of APL cells.

Vahdat et al. have also shown that basal expression of cell surface molecule CD13 on APL blasts was strongly associated with the onset of ATRA syndrome [61]. The clinical manifestations of differentiation syndrome such as unexplained fever or peripheral edema suggest that cytokines may play a role. Indeed, many studies have shown the modulation of several cytokines during the induction of ATRA therapy *in vitro* [62–64].

Relapse, which occurs in 10–15% of APL patients, has significantly affected long term treatment outcomes in APL [8, 58]. High-risk patients have an approximately 15–25% chance of relapse following ATRA and anthracycline therapy [8, 58]. Relapsed patients are likely to become resistant to conventional ATRA therapy and require other treatment methods. In the subset of patients who relapse, ATO has been shown to have remarkable clinical efficacy, with close to 85% of patients achieving complete remissions [65–68]. However, for the subset of patients that do not respond to ATO, treatment still remains an issue as attempts to overcome arsenic trioxide resistance have failed. A new synthetic retinoid Tamibarotene (Am80) with higher binding affinity to PML-RARA than ATRA has been under investigation. Tamibarotene is a potent inducer of differentiation in HL-60 and NB-4 cell lines *in vitro*; the synthetic retinoid has also shown a longer half-life when compared to ATRA [69]. Clinical studies have shown a modest effect in relapsed patients; however, statistical differences in efficacy seem to vanish at 5 years [69].

The absolute quantification of PML-RARA prior to treatment may be suggestive of the risk of relapse in APL patients undergoing therapy. Albano et al. have shown that at a 5 year follow up, patients with >209.6 PML-RARA/ng of transcript at diagnosis had a 50.3% incidence of relapse [70]. These results suggest that the absolute quantification of PML-RARA transcript may serve as a valuable prognostic indicator in patients with the susceptibility to relapse.

The majority of APL patients (~95%) present with the classic PML-RARA translocation. However, approximately 5% of patients with APL have a non-characteristic translocation; one of them being the PLZF/RARA translocation [71]. Patients with a PLZF/RARA fusion gene are less responsive to ATRA and have a poorer prognosis [71]. Non-characteristic translocations usually render patients insensitive to ATO, being unable to cause degradation of the fusion protein [71, 72]. Other chimeric proteins have also been observed including ZBTB16-RARA, NuMaRARa, NPM-RARa, and Stat5b-RARa [73]. Gallagher et al. have reported up to 40% of relapsed patients treated with ATRA show mutations in the ligand binding domain of PML-RARA [74]. *In vitro* studies as well as the response to ATRA-ATO have predominantly focused on the t (15;17) translocation.

To date STAT5b-RARA and ZBTB16-RARA are the most common variant translocations seen clinically, representing more than 39 cases that have been identified [75]. Patients harboring these two translocations do not respond adequately to ATRA and/or ATO, complicating treatment efforts [75].

Many explanations have been proposed to explain retinoic acid resistance, such as the rapid drug metabolism, or alterations of cytoplasmic retinoic acid binding proteins (CRABP). Currently, only mutations in the ligand binding domain of the nuclear hormone receptor (RARα) have been observed to mediate retinoic acid resistance [72, 73, 76]. *In vitro* studies using NB4 resistant cell lines have shown that these mutations render PML-RARA unresponsive to retinoic acid, yet the fusion protein retains its ability to bind to retinoic acid response elements inhibiting transcription [76]. Increased degradation of the PML-RARA protein has been observed to mediate retinoic acid resistance *in vitro* [37].

Resistance to arsenic has also been observed both *in vitro* and *in vivo* [72]. Resistance to arsenic trioxide has been linked to highly clustered mutations in the region of the arsenic binding site of the PML moiety of PML-RARA; the most common mutation involves a single base substitution (A216V/T) [72]. Recent evidence using *in vitro* models has shown that mutations in the B2 domain of the PML protein, which acts as a direct target for arsenic binding, mediate arsenic resistance.

In a longitudinal analysis of treatment efficacy, Zhu et al. evaluated 35 relapsed patients who were initially treated with the recommended ATRA/ATO combination for arsenic-resistant disease [77]. Of these 35 patients, 13 had arsenic-resistant disease defined as patients who did not undergo remission after arsenic induction therapy [77]. Of the 13 patients with arsenic resistant disease, 11 (~85%) eventually died [77]. Furthermore, 9 of 13 patients with arsenic resistant disease had a mutation in the PML region of the PML-RARA fusion protein [77]. No other treatment, including cytotoxic chemotherapy, was successful in treating arsenic resistance in this subset of patients.

The outcomes in treatment of relapsed APL were investigated by Lu et al. in a subset of 25 patients who initially received ATRA + ATO therapy [78]. Of these 25 patients, 8 (32%) achieved complete remission while 17 (68%) had died. Lu et al. also observed a significantly higher rate of extramedullary relapse with CNS involvement in one fifth of the patients [78]. Given that a majority of the patients were unable to achieve complete remission, the potential for secondary relapse in patients initially treated with ATRA + ATO combination therapy may require further evaluation. Overall, treatment protocols for relapsed APL remains poorly defined as many clinicians elect to use chemotherapeutic agents in addition to the combination therapy. Furthermore, many clinical trials involve the administration of chemotherapy in APL patients that are not classified as high risk.

The implications of clinical resistance to arsenic trioxide, as well as the poor prognosis of these patients, have encouraged *in vitro* studies in which leukemic cell lines are exposed to increasing levels of ATO [79]. As a result, ATO resistance can be studied *in vitro* using candidate cell line models. Zhu et al. have observed the presence of a PML mutational hot-spot (C212-S220) in ATO-resistant APL [77, 80]. Double resistant cell lines created *in vitro*, that are resistant to both ATRA and ATO have the potential to serve as important models for relapsed patients that no longer respond to the conventional treatment.

## IMPLICATIONS OF FLT3-ITD IN APL

The presence of FLT3-ITD’s is associated poorer outcomes in primary APL, however, the reason behind this was largely unknown [81]. Recent evidence using *in vitro* murine APL models, suggests that FLT3-ITD may contribute to the development of ATRA resistance [82]. FLT3-ITD’s attenuate ATRA response in APL cells and impede the degradation of the PML-RARA fusion protein, likely due to the disruption of nuclear bodies [82]. P53 signaling has also shown to be disrupted when FLT3-ITD’s are present in APL [82]. Importantly, combination treatment with ATRA/ATO has the ability to overcome ATRA resistance mediate by FLT3-ITD’s fully restoring nuclear body formation and degradation of the PML-RARA protein [82].

## ATTENUATED ARSENIC TRIOXIDE TREATMENT

An attenuated schedule of ATO in patients with APL has recently been implemented in clinical trials, where patients receive a lower cumulative dose and have fewer days of ATO treatment. Long-term follow-up results of the United Kingdom National Cancer Research Institute (NCRI) AML17 trial were recently reported [83]. Patients were randomized to receive either ATRA + attenuated ATO or anthracycline + ATRA therapy (AIDA). Patients from all risk groups were included. Patients were randomized to receive either ATRA (45 mg/m^2^) + attenuated ATO or the AIDA schedule [83]. The attenuated ATO schedule consisted of 8 weeks of induction therapy (week 1: 0.3 mg/kg on days 1–5, weeks 2–8: 0.25 mg/kg twice a week) followed by 4 weeks of consolidation therapy (week 1: 0.3 mg/kg on days 1–5, weeks 2–4:0.25 mg/kg twice a week) [83]. The AIDA schedule consisted of idarubicin (IDA) 12 mg/m^2^ on days 2,4,6, and 8 + ATRA to day 60 for induction [83]. This was followed by IDA 5 mg/m^2^ on days 1–4 + ATRA on days 1–15 (course 2), mitoxantrone 10 mg/m^2^ on days 1–5 + ATRA on days 1–15 (course 3), and IDA 12 mg/m^2^ on day 1 + ATRA on days 1–15 (course 4) [83]. Maintenance therapy was not given.

A total of 189 patients were treated with the AIDA regimen; among this cohort 33 relapsed. 32 out of 33 patients were then put on the ATRA + attenuated ATO schedule post relapse [83]. 57 high-risk patients (WBC > 10 × 10^9^/L) were included in this study [83]. No significant difference in complete remission rates or overall survival was noted between the two arms [83]. For AIDA treated patients the 5 year incidence of any relapse was 20% [83]. Among patients who became molecularly negative in the ATRA + ATO arm, none relapsed [83]. A benefit in relapse free survival (RFS) was noted in the ATO group (96% vs 86%; HR, 0.43; 95% CI, 0.18–1.03; *P* = .06) [83]. The lack of benefit in survivorship between arms may be explained by the 32 patients initially treated with the AIDA regimen who were placed on the attenuated ATO schedule following relapse.

## SYNERGISTIC EFFECTS OF ATRA & ATO

Treatment of APL patients with ATRA alone is ineffective in inducing durable remissions. The recent implementation of combination therapy with ATRA and ATO has revolutionized APL treatment leading to high cure rates. Recent studies have illuminated the mechanisms behind the synergism achieved with ATRA/ATO combination therapy [84]. APL is defined by large scale epigenetic changes and transcriptional repression of retinoic acid receptor target genes [84]. Transglutaminase 2 (*TGM2*) and retinoic acid receptor beta (*RARβ*) are two genes intrically involved in retinoic acid mediated differentiation [84]. Both genes have been shown to be heavily methylated in APL and undergo extensive histone modification during leukomegenesis [84]. Recent evidence by Huynh et al. suggests that combination treatment with ATRA and ATO leads to a sustained expression of target genes (TGM2 & RARβ) leading to terminal differentiation of NB4 promyelocytes [84].

The benefits of combination therapy as opposed to single agents are most pronounced after 96 h of treatment termination [84]. When NB4 cells are treated with a single agent alone (ATRA or ATO) differentiation markers disappear after 96 hours post treatment termination [84]. Conversely in combination treatment (ATRA+ATO) differentiation is sustained and cells express differentiation markers (CD11b) well after treatment termination [84]. Transcript levels of important differentiation genes such as TGM2, RARβ, CCL2 and ASB2 were significantly higher 96 hours post treatment with combination therapy when compared to single agents alone [84]. Combination treatment has also shown the ability to induce to demethylation in CpG islands of TGM2 and RARβ promoter regions [84].

## CONCLUSIONS

The biochemical and mechanistic research on APL over the past few decades has led to a unique understanding of this disease and the treatment options, ushering in an era of targeted therapy. Despite remarkable scientific advances in treating APL, some issues still remain, concerning high-risk patients and patients exhibiting an uncharacteristic translocation. The use of HI-60 and NB4 cell lines will continue to be beneficial for future studies on APL, since they have already shown a remarkable translational potential and will help address the therapeutic needs of patients that do not respond to conventional treatment. Further studies, addressing aspects of differentiation, nuclear body formation, and degradation of fusion protein are essential for advancing the treatment of APL and targeting it towards each affected individual. The investigation for alternative therapies for relapsed APL patients and the introduction of clear, defined treatment guidelines in each risk classified group are of particular concern to be addressed.
